# Supplementary figures and images for: RNAi-based screens uncover a potential new role for the orphan neuropeptide receptor Moody in Drosophila female germline stem cell maintenance
Source: PLoS One. 2020 Dec 11;15(12):e0243756. doi: 10.1371/journal.pone.0243756 (PMC7732368; doi:10.1371/journal.pone.0243756)

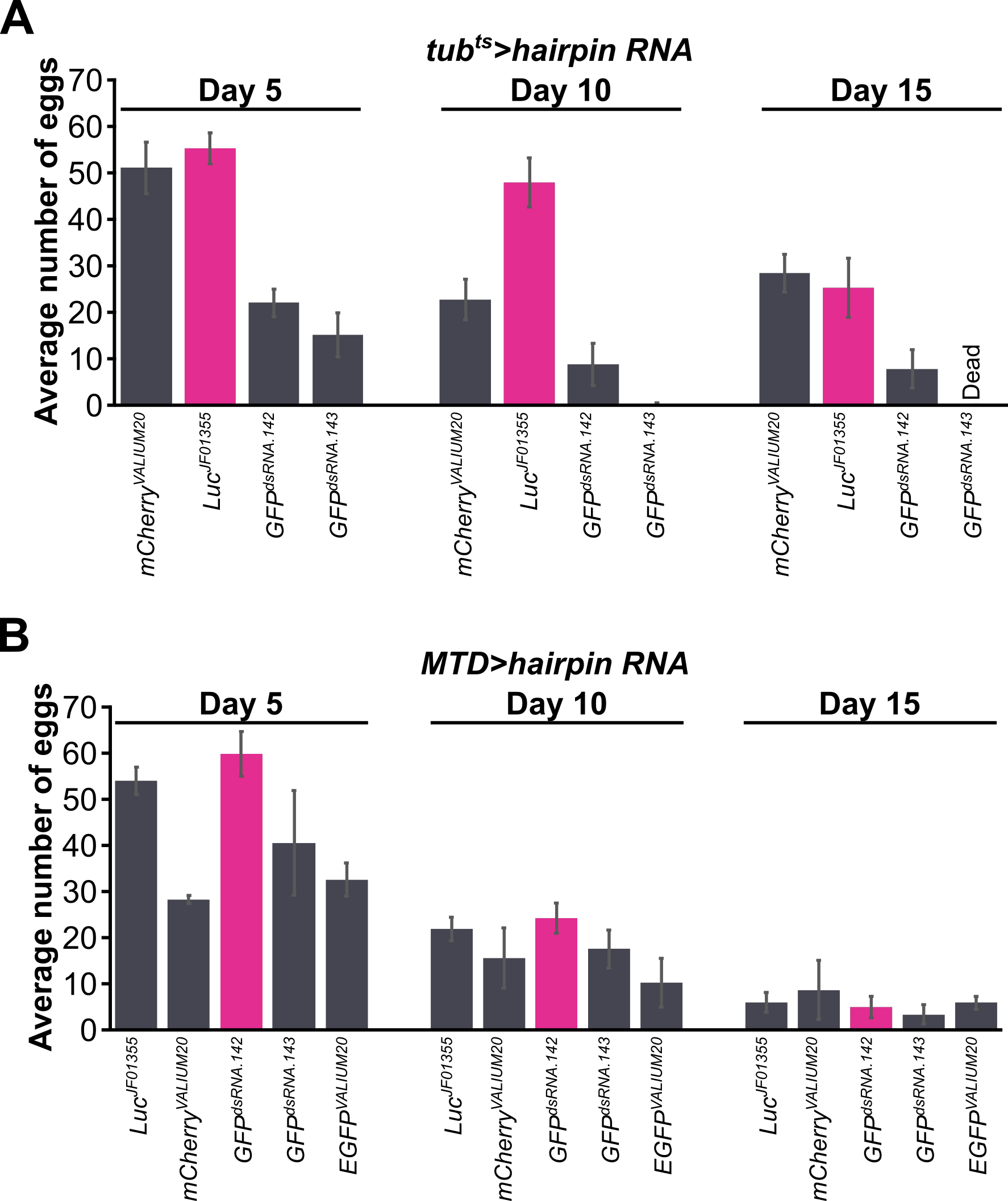

Supplement: S1 Fig — (A,B) Average number of eggs laid per female per day for females with tubts (A) or MTD-Gal4 (B) driving different control UAS-hairpin RNA transgenes, raised at 25°C, and switched to 29°C for five, 10, or 15 days. Data shown as mean±s.e.m. (TIFF) [file pone.0243756.s001.tiff]

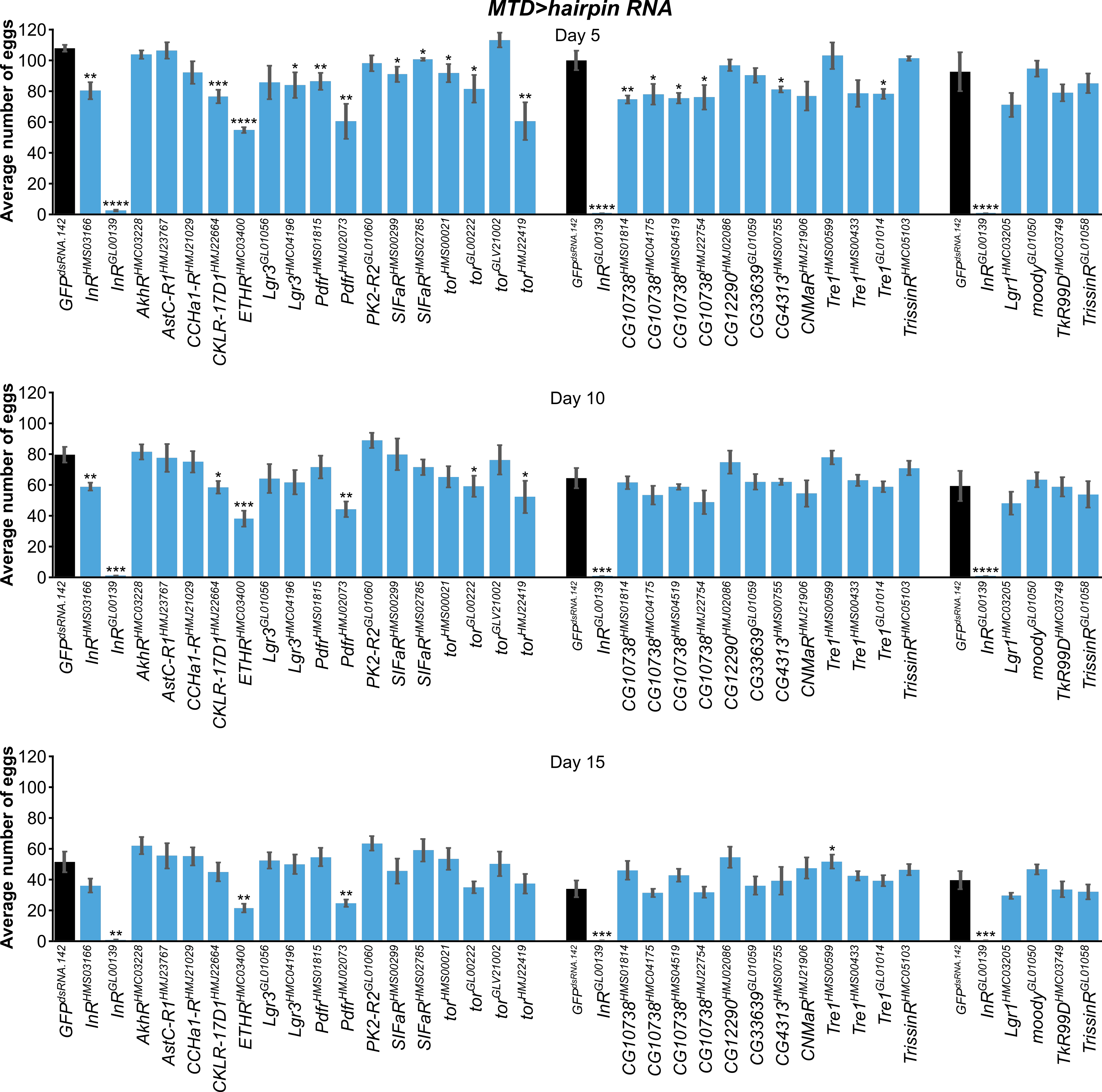

Supplement: S2 Fig — MTD was used to drive UAS-hairpin RNA against neuropeptide receptor genes, and the number of eggs laid per female per day was counted on days five, 10, and 15. MTD>GFPdsRNA.142 served as negative control. InR knockdown served as an internal control. *p<0.05; **p<0.01; ***p<0.001; ****p<0.0001, Student’s t-test. Data shown as mean±s.e.m. (TIFF) [file pone.0243756.s002.tiff]

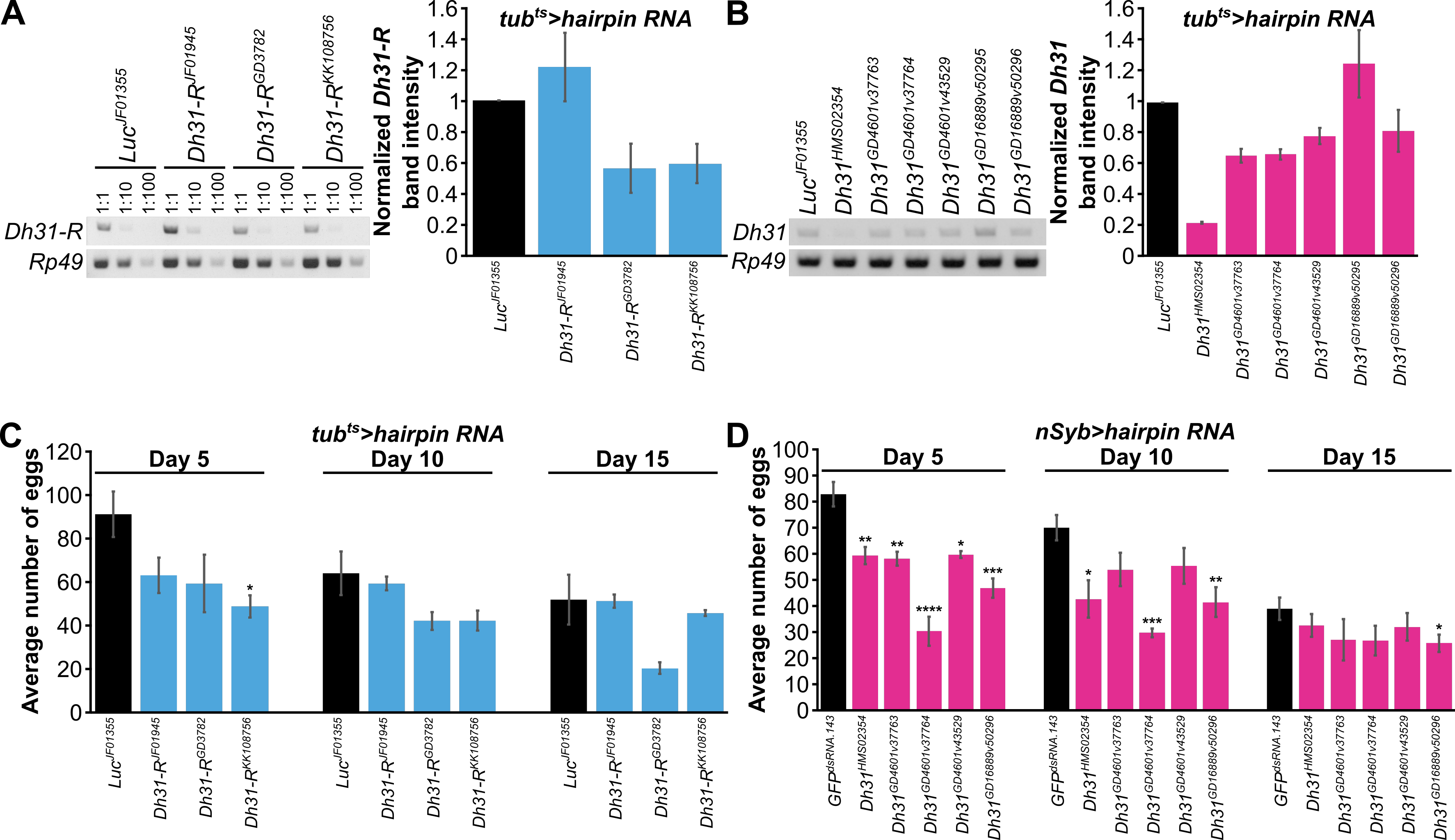

Supplement: S3 Fig — (A) Representative gel (left) and quantification (right) of RT-PCR analysis of Dh31-R transcript levels in female guts at seven days of ubiquitous somatic knockdown of Dh31-R or Luc control. Rp49 was used as a control. For each genotype, the ratio of Dh31-R band intensity (1:1 dilution) relative to Rp49 intensity (1:10 dilution) was normalized to that of tubts>LucJF01355, which was arbitrarily set at one. Three biological replicates were used for each genotype, with 10 guts per biological replicate. Gut-derived RNA was used as Dh31-R is most highly expressed in the gut in adults (www.flybase.org). (B) RT-PCR analysis of Dh31 transcript levels in female heads at seven days of ubiquitous somatic knockdown of Dh31 or Luc control. Dh31 relative to Rp49 band intensity normalized to tubts>LucJF01355 control was calculated as described in (A). Three biological replicates were used for each genotype, with 10 heads per biological replicate. Data shown as mean±s.e.m. (C) Graph showing the average number of eggs laid per female per day at five, 10, and 15 days of ubiquitous somatic knockdown of Dh31-R or Luc control. (D) Graph showing the average number of eggs laid per female per day at five, 10, and 15 days of pan-neuronal RNAi of Dh31 or Luc control. *p<0.05; **p<0.01; ***p<0.001; ****p<0.0001, Student’s t-test. Data shown as mean±s.e.m. (TIFF) [file pone.0243756.s003.tiff]

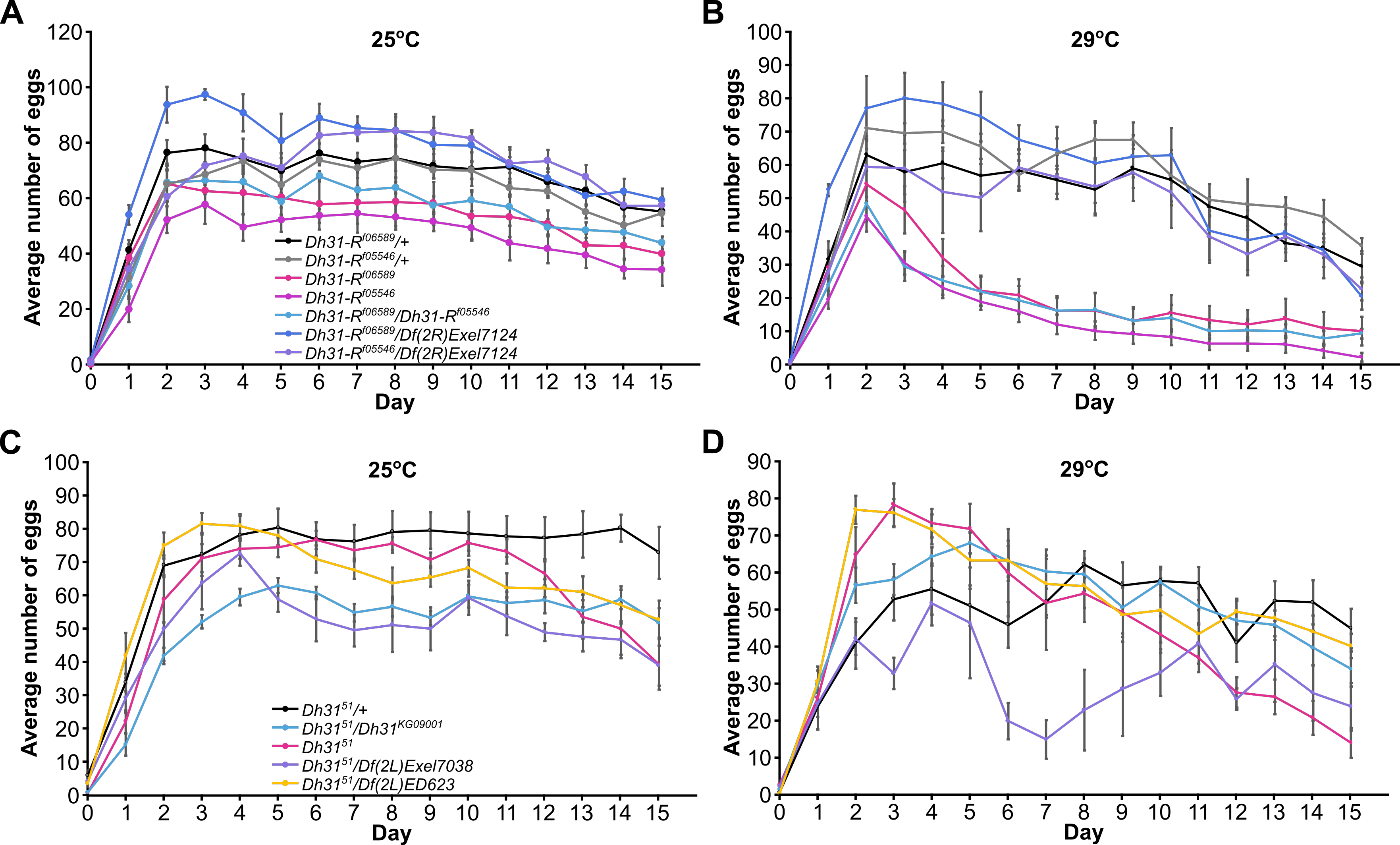

Supplement: S4 Fig — (A,B) Line graphs showing the average number of eggs laid per female per day at different days after eclosion for Dh31-R mutants and heterozygous controls at 25°C (A) or 29°C (B). Data from days five, 10, and 15 at 25°C are also shown in Fig 2G. Note that in (B), the two homozygous mutants and transheterozygous mutant lay fewer eggs at 29°C; this is likely due to linked background mutation as neither Dh31-Rf06589/Df(2R)Exel7124 nor Dh31-Rf05546/Df(2R)Exel7124 lay fewer eggs than heterozygous controls. (C,D) Line graphs showing the average number of eggs laid per female per day for Dh31 mutants and heterozygous controls at 25°C (C) or 29°C (D). Data from day five, 10, and 15 at 25°C are also shown in Fig 2H. Data shown as mean±s.e.m. (TIFF) [file pone.0243756.s004.tiff]

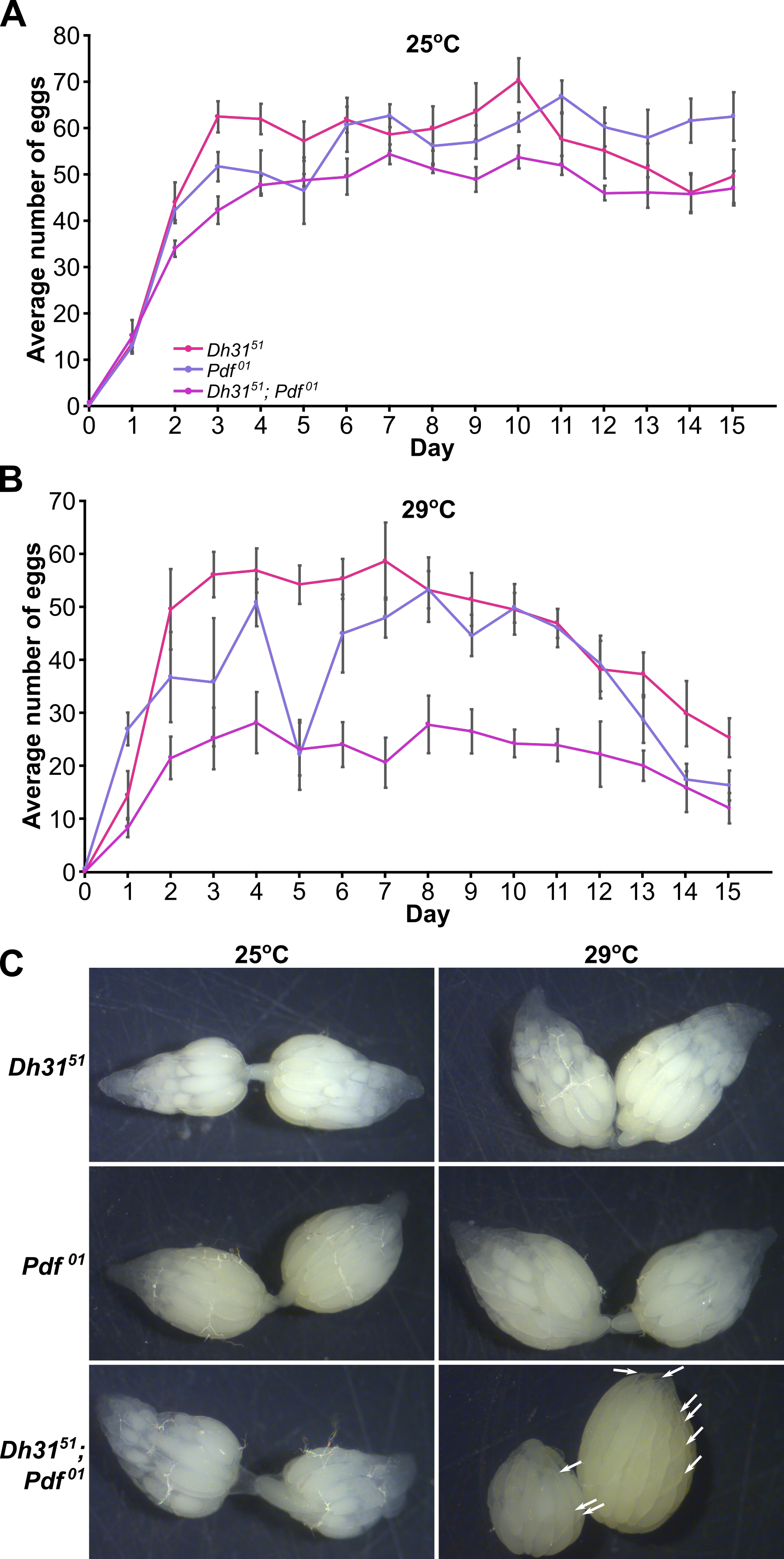

Supplement: S5 Fig — (A,B) Line graphs showing the average number of eggs laid per female per day at different days after eclosion for Dh3151 homozygous, Pdf01 homozygous, or Dh3151; Pdf01 double homozygous females at 25°C (A) or 29°C (B). Data shown as mean±s.e.m. (C) Examples of ovaries from Dh3151, Pdf01, and Dh3151; Pdf01 5-day-old females at 25°C (left) or 29°C (right). Arrows point to multiple dorsal appendages to indicate examples of accumulated mature oocytes. (TIFF) [file pone.0243756.s005.tiff]

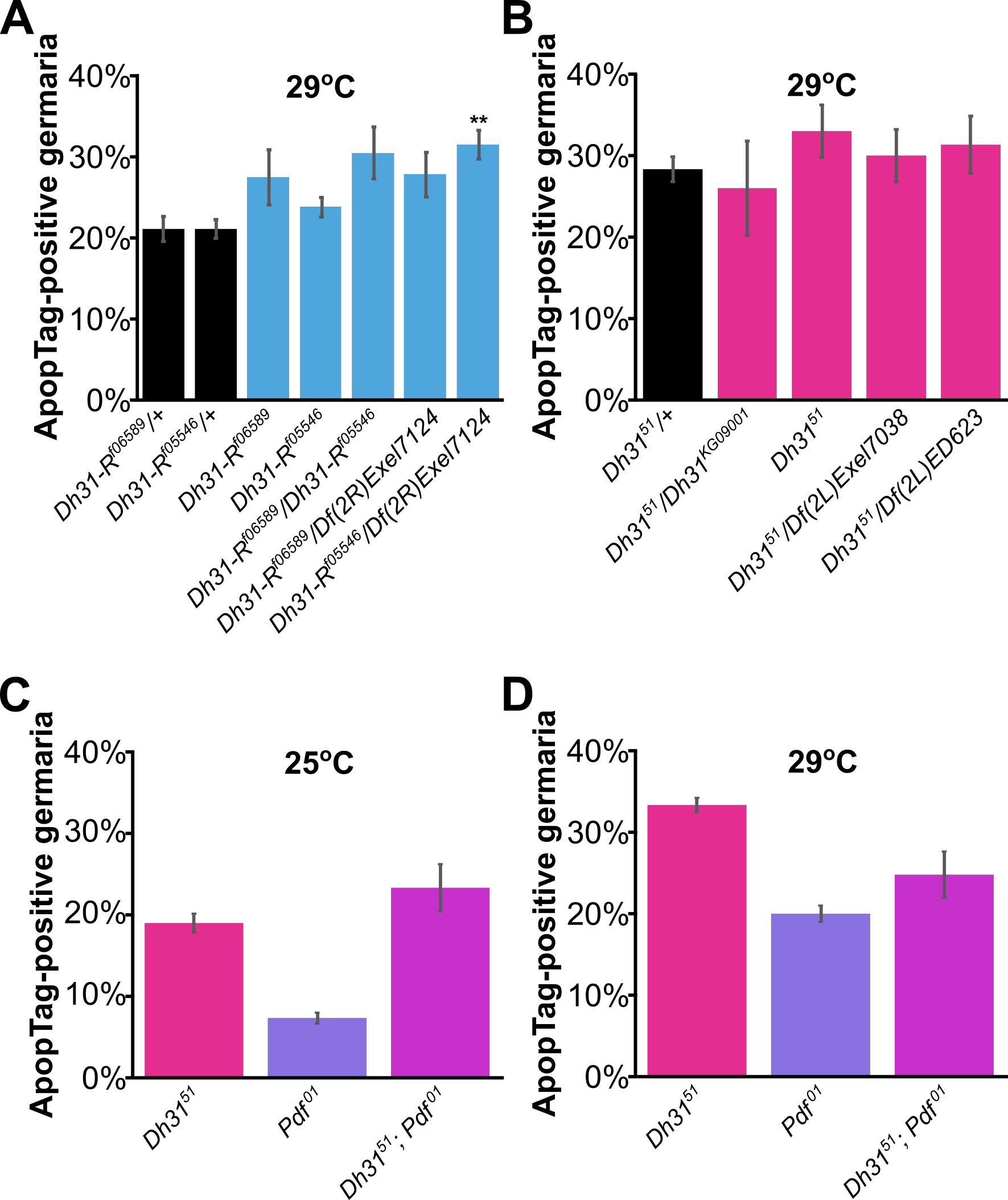

Supplement: S6 Fig — Percent of germaria showing ApopTag-positive germline cysts in five-day old Dh31-R mutant (A), Dh31 mutant (B), or Dh3151; Pdf01 females at 25°C (C) or 29°C (D). Three biological replicates per genotype, 100 germaria per replicate. **p<0.01, Student’s t-test. Data shown as mean±s.e.m. (TIFF) [file pone.0243756.s006.tiff]

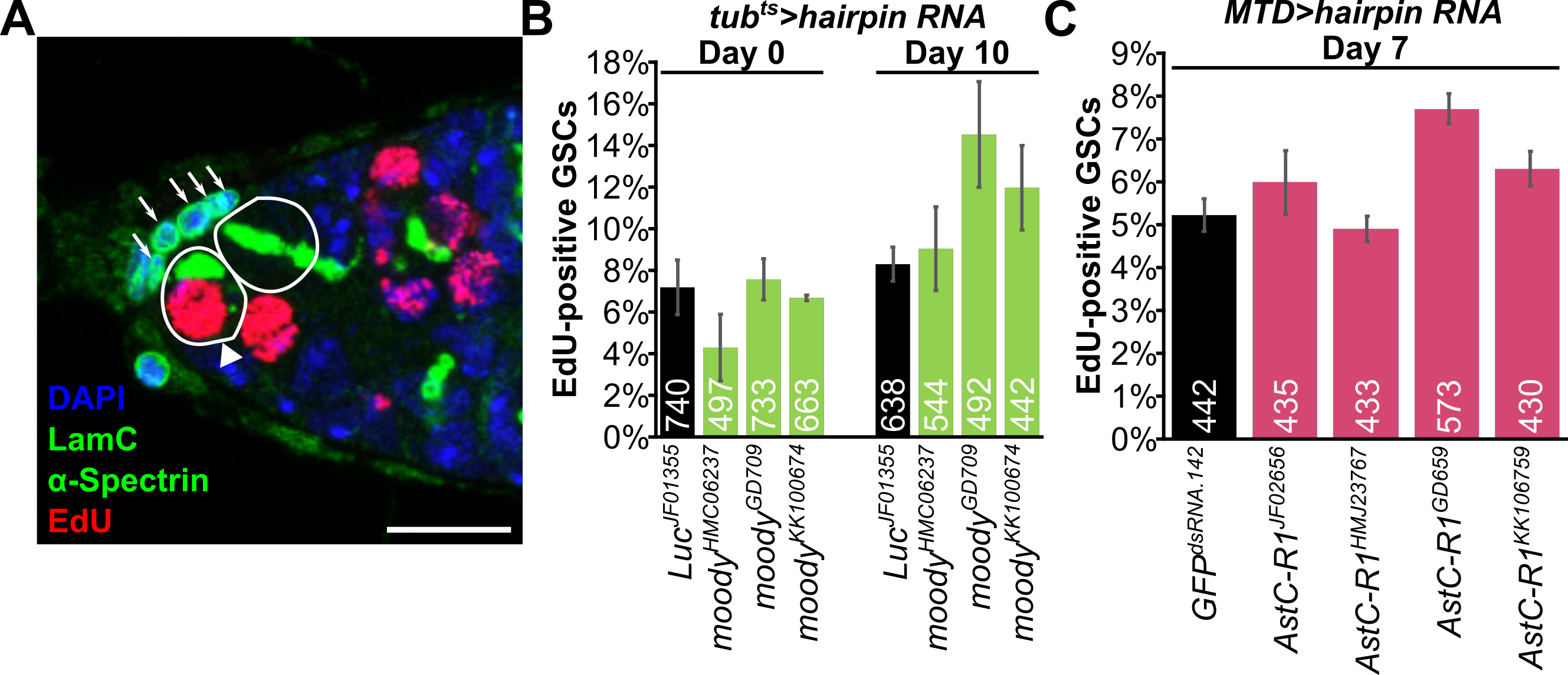

Supplement: S7 Fig — (A) Example of germarium from MTD>GFPdsRNA.142 female at 7 days of GFP knockdown showing one EdU-positive GSC (arrowhead) and one EdU-negative GSC. DAPI (blue) labels nuclei. LamC (green), nuclear lamina of cap cells; α-Spectrin (green), fusome; EdU (red), S-phase marker. Cap cells, arrows; GSCs, solid outlines. Scale bar, 10 μm. (B,C) Graphs showing the average percentage of EdU-positive GSCs at zero or 10 days of somatic knockdown of moody or Luc control (B) or at seven days of germline knockdown of AstC-R1 or GFP (C). Two biological replicates for moodyHMC06237, GFPdsRNA.142, and all Astc-R1 RNAi genotypes, and three biological replicates for all other genotypes. The total number of GSCs analyzed are indicated inside bars. (TIFF) [file pone.0243756.s007.tiff]

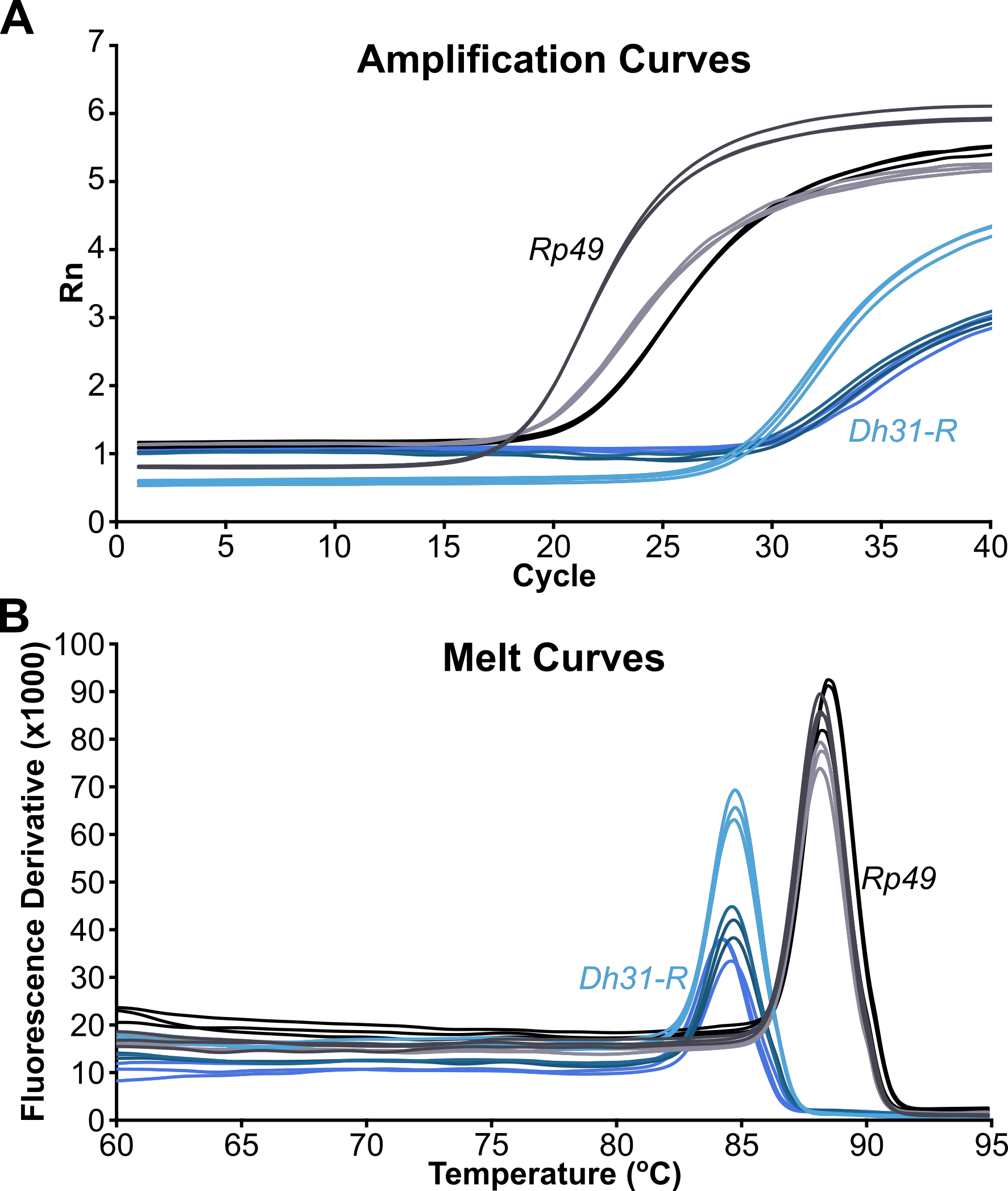

Supplement: S8 Fig — (A) Amplification curves for three biological replicates of Dh31-Rf05546/+, with three technical replicates per biological sample, plotting Rn versus cycle number, with amplification for Dh31-R shown in shades of blue and Rp49 in shades of gray. (B) Melt curves for the same sample of Dh31-Rf05546/+ plotting the derivative of fluorescence intensity versus temperature, with Dh31-R products in shades of blue and Rp49 products in shades of gray. Each line represents one technical replicate of a biological replicate. Biological replicates were collected at different times, and qPCR was performed separately. (TIFF) [file pone.0243756.s008.tiff]
