# Supplementary material for: RNAi-based screens uncover a potential new role for the orphan neuropeptide receptor Moody in Drosophila female germline stem cell maintenance
Source: PLoS One. 2020 Dec 11;15(12):e0243756. doi: 10.1371/journal.pone.0243756 (PMC7732368; doi:10.1371/journal.pone.0243756)
Supplement: S1 Table — (PDF) [file pone.0243756.s009.pdf]

S1 Table. Transgenic *Drosophila* lines used in this study.

Name	Genotype	Source	Reference
**Gal4 Drivers**			
hh-Gal4 (GSC niche)	w^{1118}; *P{GMR28E03-GAL4}attP2*	Erika Matunis (BDSC 45546)	[1]
MTD (germline)	*P{otu-GAL4::VP16.R}1*, w^{*}; *P{GAL4-nos.NGT}40; P{GAL4::VP16-nos.UTR}*$CG6325^{MVD1}$	BDSC 31777	[2]
nSyb-Gal4 (neurons)	*nSyb-Gal4/TM6.Sb*	Mark Wu	[3]
tub^{ts} (soma)	$tubP\text{-}Gal80^{ts}$*/CyO; tubP-Gal4/TM6b*		[4]
nos-Gal4 (germline)	w^{*}; *PBac{GreenEye.nosGAL4}Dmel2*	BDSC 32179	[5]
**Mutant and Deficiency Lines**			
$Dh31\text{-}R^{f05546}$	w^{1118}; *PBac{WH}*$Dh31\text{-}R^{f05546}$	BDSC 85703	[6,7]
$Dh31\text{-}R^{f06589}$	w^{1118}; *PBac{WH}*$Dh31\text{-}R^{f06589}$*/CyO*	Fumika Hamada (BDSC 85728)	[6,7]
Df(2R)Exel7124	w^{1118}; *Df(2R)Exel7124/CyO*	BDSC 7872	[8]
$Dh31^{KG09001}$	y^{1}; *P{SUPor-P}*$Dh31^{KG09001}$	BDSC 16474	[9,10]
$Dh31^{51}$	$Dh31^{51}$	Fumika N. Hamada	[11]
Df(2L)ED623	w^{1118}; *Df(2L)ED623, P{3'.RS5+3.3'}ED623/SM6a*	BDSC 8930	[12]
Df(2L)Exel7038	w^{1118}; *Df(2L)Exel7038/CyO*	BDSC 7809	[8]
Pdf^{01}	*w*; Pdf^{01}	Fumika N. Hamada	[6,13]
$Dh31^{51}$; Pdf^{01}	*w*; $Dh31^{\#51}$; Pdf^{01}	Fumika N. Hamada	[6]

**Control dsRNA Lines**			
$EGFP^{VALIUM20}$	y^{1} sc^{*} v^{1}; *P{VALIUM20-EGFP}attP2*	BDSC 35782	[14]
$GFP^{dsRNA.142}$	w^{1118}; *P{UAS-GFP.dsRNA.R}142*	BDSC 9330	[15]
$GFP^{dsRNA.143}$	w^{1118}; *P{UAS-GFP.dsRNA.R}143*	BDSC 9331	[15]
$Luciferase^{JF01355}$	y^{1} v^{1}; *P{TRiP.JF01355}attP2*	BDSC 31603	[14]
$mCherry^{VALIUM20}$	y^{1} sc^{*} v^{1} sev^{21}; *P{VALIUM20-mCherry}attP2*	BDSC 35785	[14]
**Neuropeptide dsRNA Lines**			
$Akh^{HMS00477}$	y^{1} sc^{*} v^{1} sev^{21}; *P{TRiP.HMS00477}attP2*	BDSC 34960	[14]
$amn^{JF01814}$	y^{1} v^{1}; *P{TRiP.JF01814}attP2*	BDSC 25797	[14]
$AstA^{JF01905}$	y^{1} v^{1}; *P{TRiP.JF01905}attP2*	BDSC 25866	[14]
$AstC^{JF01907}$	y^{1} v^{1}; *P{TRiP.JF01907}attP2*	BDSC 25868	[14]
$AstCC^{HMJ21833}$	y^{1} v^{1}; *P{TRiP.HMJ21833}attP40*	BDSC 57825	[14]
$Burs^{JF02260}$	y^{1} v^{1}; *P{TRiP.JF02260}attP2*	BDSC 26719	[14]
$Capa^{JF02981}$	y^{1} v^{1}; *P{TRiP.JF02981}attP2/TM3*, Sb^{1}	BDSC 28345	[14]
$CCAP^{JF03248}$	y^{1} v^{1}; *P{TRiP.JF03248}attP2*	BDSC 29569	[14]
$CCHa1^{HMC04879}$	y^{1} sc^{*} v^{1} sev^{21}; *P{TRiP.HMC04879}attP40*	BDSC 57562	[14]
$CCHa2^{HMC04565}$	y^{1} sc^{*} v^{1} sev^{21}; *P{TRiP.HMC04565}attP40*	BDSC 57183	[14]
$CNMa^{KK110796}$	*P{KK110796}VIE-260B*	VDRC 104599 KK	[16]
$Crz^{JF02023}$	y^{1} v^{1}; *P{TRiP.JF02023}attP2*	BDSC 25999	[14]
$Dh31^{HMS02354}$	y^{1} sc^{*} v^{1} sev^{21}; *P{TRiP.HMS02354}attP2/TM3*, Sb^{1}	BDSC 41957	[14]
$Dh31^{GD4601v37763}$	w^{1118}; *P{GD4601}v37763*	VDRC 37763 GD	[16]

$Dh31^{GD4601v37764}$	w^{1118}; P{GD4601}v37764	VDRC 37764 GD	[16]
$Dh31^{GD4601v43529}$	w^{1118}; P{GD4601}v43529/TM3	VDRC 43529 GD	[16]
$Dh31^{GD16889v50295}$	w^{1118} P{GD16889}v50295	VDRC 50295 GD	[16]
$Dh31^{GD16889v50296}$	w^{1118}; P{GD16889}v50296/TM3	VDRC 50296 GD	[16]
$Dh44^{JF01822}$	y^1 v^1; P{TRiP.JF01822}attP2	BDSC 25804	[14]
$Dsk^{JF01908}$	y^1 v^1; P{TRiP.JF01908}attP2	BDSC 25869	[14]
$Eh^{JF02143}$	y^1 v^1; P{TRiP.JF02143}attP2	BDSC 26244	[14]
ETH^{GD5964}	w^{1118} P{GD5964}v18825	VDRC 18825 GD	[16]
$FMRFa^{HMJ22167}$	y^1 v^1; P{TRiP.HMJ22167}attP40	BDSC 58197	[14]
$Hug^{JF03122}$	y^1 v^1; P{TRiP.JF03122}attP2	BDSC 28705	[14]
$ITP^{JF01817}$	y^1 v^1; P{TRiP.JF01817}attP2	BDSC 25799	[14]
$Lk^{JF01816}$	y^1 v^1; P{TRiP.JF01816}attP2	BDSC 25798	[14]
$Mip^{HMS02244}$	y^1 sc^* v^1 sev^{21}; P{TRiP.HMS02244}attP2	BDSC 41680	[14]
$Ms^{JF02144}$	y^1 v^1; P{TRiP.JF02144}attP2/TM3, Sb^1	BDSC 26245	[14]
$NPF^{JF02555}$	y^1 v^1; P{TRiP.JF02555}attP2	BDSC 27237	[14]
$Nplp1^{JF01911}$	y^1 v^1; P{TRiP.JF01911}attP2	BDSC 25872	[14]
$Nplp2^{HMJ21350}$	y^1 v^1; P{TRiP.HMJ21350}attP40	BDSC 53967	[14]
$Nplp3^{JF03188}$	y1 v1; P{TRiP.JF03188}attP2	BDSC 28760	[14]
$Nplp4^{JF03221}$	y^1 v^1; P{TRiP.JF03221}attP2	BDSC 28793	[14]
$Pburs^{HMC04211}$	y^1 sc^* v^1 sev^{21}; P{TRiP.HMC04211}attP40	BDSC 55924	[14]
$Pdf^{JF01820}$	y^1 v^1; P{TRiP.JF01820}attP2	BDSC 25802	[14]

$Proc^{JF03249}$	y^1 v^1; *P{TRiP.JF03249}attP2*	BDSC 29570	[14]
$Ptth^{JF01349}$	y^1 v^1; *P{TRiP.JF01349}attP2/TM3, Ser*^{1}	BDSC 31380	[14]
$SIFa^{JF03364}$	y^1 v^1; *P{TRiP.JF03364}attP2*	BDSC 29428	[14]
$sNPF^{JF01906}$	y^1 v^1; *P{TRiP.JF01906}attP2*	BDSC 25867	[14]
$SP^{JF02022}$	y^1 v^1; *P{TRiP.JF02022}attP2*	BDSC 25998	[14]
$Tk^{JF01818}$	y^1 v^1; *P{TRiP.JF01818}attP2*	BDSC 25800	[14]
**Neuropeptide Receptor and GPCR dsRNA Lines**			
$AkhR^{HMC03228}$	y^1 v^1; *P{TRiP.HMC03228}attP40*	BDSC 51710	[14]
$AkhR^{JF03256}$	y^1 v^1; *P{TRiP.JF03256}attP2*	BDSC 29577	[14]
$AstA\text{-}R1^{JF02578}$	y^1 v^1; *P{TRiP.JF02578}attP2*	BDSC 27280	[14]
$AstA\text{-}R2^{JF01955}$	y^1 v^1; *P{TRiP.JF01955}attP2*	BDSC 25935	[14]
$AstC\text{-}R1^{GD659}$	w^{1118}; *P{GD659}v13560/TM3*	VDRC 13560 GD	[16]
$AstC\text{-}R1^{HMJ23767}$	y^1 v^1; *P{TRiP.HMJ23767}attP40/CyO*	BDSC 62372	[14]
$AstC\text{-}R1^{JF02656}$	y^1 v^1; *P{TRiP.JF02656}attP2/TM3, Sb*^{1}	BDSC 27506	[14]
$AstC\text{-}R1^{KK106759}$	*P{KK106759}VIE-260B*	VDRC 110739 KK	[16]
$AstC\text{-}R2^{GD17055}$	w^{1118}; *P{GD17055}v50000/TM3*	VDRC 50000 GD	[16]
$AstC\text{-}R2^{GL01063}$	y^1 sc^* v^1 sev^{21}; *P{TRiP.GL01063}attP2*	BDSC 36888	[14]
$AstC\text{-}R2^{JF01960}$	y^1 v^1; *P{TRiP.JF01960}attP2*	BDSC 25940	[14]
$AstC\text{-}R2^{KK102954}$	*P{KK102954}VIE-260B*	VDRC 106146 KK	[16]
$CapaR^{JF02577}$	y^1 v^1; *P{TRiP.JF02577}attP2*	BDSC 27275	[14]
$CCAP\text{-}R^{JF01338}$	y^1 v^1; *P{TRiP.JF01338}attP2*	BDSC 31490	[14]

CCHa1-R^{GD616}	*w^{1118}; P{GD616}v1678/TM3*	VDRC 1678 GD	[16]
CCHa1-R^{HMJ21029}	*y^1 v^1; P{TRiP.HMJ21029}attP40*	BDSC 51168	[14]
CCHa1-R^{JF02748}	*y^1 v^1; P{TRiP.JF02748}attP2*	BDSC 27669	[14]
CCHa1-R^{KK112051}	*P{KK112051}VIE-260B*	VDRC 103055 KK	[16]
CCKLR-17D1^{GD730v7231}	*w^{1118}; P{GD730}v7231/TM3*	VDRC 7231 GD	[16]
CCKLR-17D1^{HMJ22664}	*y^1 v^1; P{TRiP.HMJ22664}attP40*	BDSC 60405	[14]
CCKLR-17D1^{JF02644}	*y^1 v^1; P{TRiP.JF02644}attP2*	BDSC 27494	[14]
CCKLR-17D1^{KK108482}	*P{KK108482}VIE-260B*	VDRC 100760 KK	[16]
CCKLR-17D3^{GD731}	*w^{1118}; P{GD731}v1815*	VDRC 1815 GD	[16]
CCKLR-17D3^{GD3431}	*w^{1118}; P{GD3431}v9154/TM3*	VDRC 9154 GD	[16]
CCKLR-17D3^{JF02968}	*y^1 v^1; P{TRiP.JF02968}attP2/TM3, Sb^1*	BDSC 28333	[14]
CCKLR-17D3^{KK110484}	*P{KK110484}VIE-260B*	VDRC 102039 KK	[16]
CG10738^{HM05067}	*y^1 v^1; P{TRiP.HM05067}attP2*	BDSC 28580	[14]
CG10738^{HMC04175}	*y^1 sc^* v^1 sev^{21}; P{TRiP.HMC04175}attP2*	BDSC 55896	[14]
CG10738^{HMJ22754}	*y^1 v^1; P{TRiP.HMJ22754}attP40*	BDSC 60439	[14]
CG10738^{HMS01814}	*y^1 v^1; P{TRiP.HMS01814}attP2*	BDSC 38346	[14]
CG10738^{HMS04519}	*y^1 sc^* v^1 sev^{21}; P{TRiP.HMS04519}attP40*	BDSC 57318	[14]
CG12290^{HM05266}	*y^1 sc^* v^1 sev^{21}; P{TRiP.HM05266}attP2*	BDSC 31873	[14]
CG12290^{HMJ02086}	*y^1 v^1; P{TRiP.HMJ02086}attP40*	BDSC 42520	[14]
CG13229^{JF03355}	*y^1 v^1; P{TRiP.JF03355}attP2*	BDSC 29419	[14]
CG13575^{JF01845}	*y^1 v^1; P{TRiP.JF01845}attP2*	BDSC 25827	[14]

$CG13995^{JF02275}$	y^1 v^1; P{TRiP.JF02275}attP2	BDSC 26733	[14]
$CG13995^{KK112491}$	P{KK112491}VIE-260B	VDRC 102804 KK	[16]
$CG30340^{JF03067}$	y^1 v^1; P{TRiP.JF03067}attP2	BDSC 28652	[14]
$CG33639^{GD15023}$	w^{1118}; P{GD15023}v29644	VDRC 29644 GD	[16]
$CG33639^{GL01059}$	y^1 sc^* v^1 sev^{21}; P{TRiP.GL01059}attP2	BDSC 36826	[14]
$CG33639^{JF03029}$	y^1 v^1; P{TRiP.JF03029}attP2	BDSC 28614	[14]
$CG33639^{KK110055}$	P{KK110055}VIE-260B	VDRC 108753 KK	[16]
$CG4313^{HMS00755}$	y^1 sc^* v^1 sev^{21}; P{TRiP.HMS00755}attP2/TM3, Sb^1	BDSC 31960	[14]
$CNMaR^{HMJ21906}$	y^1 v^1; P{TRiP.HMJ21906}attP40	BDSC 57859	[14]
$CrzR^{GL00706}$	y^1 v^1; P{TRiP.GL00706}attP2/TM3, Sb^1	BDSC 42751	[14]
$CrzR^{JF02042}$	y^1 v^1; P{TRiP.JF02042}attP2	BDSC 26017	[14]
$Dh31\text{-}R^{GD3782}$	w^{1118}; P{GD3782}v8777	VDRC 8777 GD	[16]
$Dh31\text{-}R^{JF01945}$	y^1 v^1; P{TRiP.JF01945}attP2/TM3, Sb^1	BDSC 25925	[14]
$Dh31\text{-}R^{KK108756}$	P{KK108756}VIE-260B	VDRC 101995 KK	[16]
$Dh44\text{-}R1^{JF03208}$	y^1 v^1; P{TRiP.JF03208}attP2	BDSC 28780	[14]
$Dop1R1^{HM04077}$	y^1 v^1; P{TRiP.HM04077}attP2	BDSC 31765	[14]
$Dop1R1^{HMC02344}$	y^1 sc^* v^1 sev^{21}; P{TRiP.HMC02344}attP2/TM3, Sb^1	BDSC 55239	[14]
$Dop1R1^{HMC05220}$	y^1 sc^* v^1 sev^{21}; P{TRiP.HMC05200}attP40	BDSC 62193	[14]
$Dop2R^{HMC02988}$	y^1 v^1; P{TRiP.HMC02988}attP40	BDSC 50621	[14]
$Dop2R^{JF02025}$	y^1 v^1; P{TRiP.JF02025}attP2	BDSC 26001	[14]
$ETHR^{GD697}$	w^{1118}; P{GD697}v42716	VDRC 42716 GD	[16]

$ETHR^{HMC03400}$	y^{1} sc^{*} v^{1} sev^{21}; P{TRiP.HMC03400}attP2/TM3, Sb^{1} Ser^{1}	BDSC 51828	[14]
$ETHR^{JF03211}$	y^{1} v^{1}; P{TRiP.JF03211}attP2	BDSC 28783	[14]
$ETHR^{KK108855}$	P{KK108855}VIE-260B	VDRC 101996 KK	[16]
$FMRFaR^{JF01879}$	y^{1} v^{1}; P{TRiP.JF01879}attP2	BDSC 25858	[14]
hec^{GD724}	w^{1118}; P{GD724}v7223/TM3	VDRC 7223 GD	[16]
$hec^{JF03302}$	y^{1} v^{1}; P{TRiP.JF03302}attP2	BDSC 29623	[14]
$InR^{GL00139}$	y^{1} sc^{*} v^{1} sev^{21}; P{TRiP.GL00139}attP2	BDSC 35251	[14]
$InR^{HMS03166}$	y^{1} v^{1}; P{TRiP.HMS03166}attP40	BDSC 51518	[14]
$InR^{JF01482}$	y^{1} v^{1}; P{TRiP.JF01482}attP2	BDSC 31037	[14]
$Lgr1^{HMC03205}$	y^{1} sc^{*} v^{1} sev^{21}; P{TRiP.HMC03205}attP2	BDSC 51465	[14]
$Lgr1^{JF02659}$	y^{1} v^{1}; P{TRiP.JF02659}attP2	BDSC 27509	[14]
$Lgr3^{GL01056}$	y^{1} sc^{*} v^{1} sev^{21}; P{TRiP.GL01056}attP2/TM3, Sb^{1}	BDSC 36887	[14]
$Lgr3^{HMC04196}$	y^{1} sc^{*} v^{1} sev^{21}; P{TRiP.HMC04196}attP40	BDSC 55910	[14]
$Lgr3^{JF03217}$	y^{1} v^{1}; P{TRiP.JF03217}attP2	BDSC 28789	[14]
$Lgr4^{GD725}$	w^{1118}; P{GD725}v7226	VDRC 7226 GD	[16]
$Lgr4^{JF03070}$	y^{1} v^{1}; P{TRiP.JF03070}attP2	BDSC 28655	[14]
$Lgr4^{KK106305}$	P{KK106305}VIE-260B	VDRC 108915 KK	[16]
$Lgr4^{KK112141}$	P{KK112141}VIE-260B	VDRC 102681 KK	[16]
$Lkr^{JF01956}$	y^{1} v^{1}; P{TRiP.JF01956}attP2	BDSC 25936	[14]
$mAChR\text{-}C^{GD717}$	w^{1118}; P{GD717}v11465	VDRC 11465 GD	[16]
$mAChR\text{-}C^{HMJ23139}$	y^{1} v^{1}; P{TRiP.HMJ23139}attP40	BDSC 61306	[14]

mAChR-C^{JF03291}	*y^1 v^1; P{TRiP.JF03291}attP2*	BDSC 29612	[14]
mGluR^{HMS00191}	*y^1 sc^* v^1 sev^{21}; P{TRiP.HMS00191}attP2*	BDSC 34872	[14]
mGluR^{HMS02201}	*y^1 v^1; p{TRiP.HMS02201}attP40*	BDSC 41668	[14]
mGluR^{JF01958}	*y^1 v^1; P{TRiP.JF01958}attP2*	BDSC 25938	[14]
moody^{GD709}	*w^{1118}; P{GD709}v1800*	VDRC 1800 GD	[16]
moody^{GL01050}	*y^1 sc^* v^1 sev^{21}; P{TRiP.GL01050}attP2*	BDSC 36821	[14]
moody^{HMC06237}	*y^1 sc^* v^1 sev^{21}; P{TRiP.HMC06237}attP2*	BDSC 66326	[14]
moody^{KK100674}	*P{KK100674}VIE-260B*	VDRC 109601 KK	[16]
MsR1^{JF02680}	*y^1 v^1; P{TRiP.JF02680}attP2*	BDSC 27529	[14]
MsR2^{JF01850}	*y^1 v^1; P{TRiP.JF01850}attP2*	BDSC 25832	[14]
mtt^{HMS00367}	*y^1 sc^* v^1 sev^{21}; P{TRiP.HMS00367}attP2*	BDSC 32376	[14]
mtt^{HMS02793}	*y^1 sc^* v^1 sev^{21}; P{TRiP.HMS02793}attP40*	BDSC 44076	[14]
NPFR^{JF01959}	*y^1 v^1; P{TRiP.JF01959}attP2*	BDSC 25939	[14]
Pdfr^{HMJ02073}	*y^1 v^1; P{TRiP.HMJ02073}attP40*	BDSC 42508	[14]
Pdfr^{HMS01815}	*y^1 v^1; P{TRiP.HMS01815}attP40*	BDSC 38347	[14]
PK1-R^{JF02690}	*y^1 v^1; P{TRiP.JF02690}attP2*	BDSC 27539	[14]
PK1-R^{KK106944}	*P{KK106944}VIE-260B*	VDRC 101115 KK	[16]
PK2-R1^{JF03303}	*y^1 v^1; P{TRiP.JF03303}attP2*	BDSC 29624	[14]
PK2-R2^{GL01060}	*y^1 sc^* v^1 sev^{21}; P{TRiP.GL01060}attP2*	BDSC 36827	[14]
PK2-R2^{JF03209}	*y^1 v^1; P{TRiP.JF03209}attP2*	BDSC 28781	[14]
PK2-R2^{KK106245}	*P{KK106245}VIE-260B*	VDRC 100927 KK	[16]

Proc-R^{JF03350}	*y^1 v^1; P{TRiP.JF03350}attP2*	BDSC 29414	[14]
rk^{JF02678}	*y^1 v^1; P{TRiP.JF02678}attP2*	BDSC 31958	[14]
SIFaR^{HMS00299}	*y^1 sc^* v^1 sev^{21}; P{TRiP.HMS00299}attP2*	BDSC 34947	[14]
SIFaR^{HMS02785}	*y^1 sc^* v^1 sev^{21}; P{TRiP.HMS02785}attP40*	BDSC 44068	[14]
SIFaR^{JF01849}	*y^1 v^1; P{TRiP.JF01849}attP2*	BDSC 25831	[14]
sNPFR^{JF02657}	*y^1 v^1; P{TRiP.JF02657}attP2*	BDSC 27507	[14]
Tkr86C^{GD681}	*w^{1118}; P{GD681}v13392*	VDRC 13392 GD	[16]
TkR86C^{JF02160}	*y^1 v^1; P{TRiP.JF02160}attP2*	BDSC 31884	[14]
TkR86C^{KK103061}	*P{KK103061}VIE-260B*	VDRC 107090 KK	[16]
TkR99D^{HMC03749}	*y^1 sc^* v^1 sev^{21}; P{TRiP.HMC03749}attP40*	BDSC 55732	[14]
TkR99D^{JF02663}	*y^1 v^1; P{TRiP.JF02663}attP2*	BDSC 27513	[14]
tor^{GLO00222}	*y^1 sc^* v^1 sev^{21}; P{TRiP.GL00222}attP2*	BDSC 35316	[14]
tor^{GLV21002}	*y^1 sc^* v^1 sev^{21}; P{TRiP.GLV21002}attP2*	BDSC 35639	[14]
tor^{HMJ22419}	*y^1 v^1; P{TRiP.HMJ22419}attP40*	BDSC 58312	[14]
tor^{HMS00021}	*y^1 v^1; P{TRiP.HMS00021}attP2*	BDSC 33627	[14]
Tre1^{GD715v7219}	*w^{1118}; P{GD715}v7219*	VDRC 7219 GD	[16]
Tre1^{GL01014}	*y^1 sc^* v^1 sev^{21}; P{TRiP.GL01014}attP40/CyO*	BDSC 38925	[14]
Tre1^{HMS00433}	*y^1 sc^* v^1 sev^{21}; P{TRiP.HMS00433}attP2*	BDSC 34956	[14]
Tre1^{HMS00599}	*y^1 sc^* v^1 sev^{21}; P{TRiP.HMS00599}attP2/TM3, Sb^1*	BDSC 33718	[14]
Tre1^{JF02751}	*y^1 v^1; P{TRiP.JF02751}attP2*	BDSC 27672	[14]
TrissinR^{GD927v7886}	*w^{1118}; P{GD927}v7886*	VDRC 7886 GD	[16]

TrissinR^{GD928v42758}	*w*^{1118}*; P{GD928}v42758*	VDRC 42758 GD	[16]
TrissinR^{GL01058}	*y*^{1} *sc** *v*^{1} *sev*^{21}*; P{TRiP.GL01058}attP2*	BDSC 36825	[14]
TrissinR^{HMC05103}	*y*^{1} *sc** *v*^{1} *sev*^{21}*; P{TRiP.HMC05103}attP40*	BDSC 60109	[14]
TrissinR^{KK107157}	*P{KK107157}VIE-260B*	VDRC 107943 KK	[16]

Supplemental References

1. Jenett A, Rubin GM, Ngo TTB, Shepherd D, Murphy C, Dionne H, et al. A GAL4-Driver Line Resource for Drosophila Neurobiology. Cell Rep. 2012;2: 991–1001. doi:10.1016/j.celrep.2012.09.011
2. Mazzalupo S, Cooley L. Illuminating the role of caspases during Drosophila oogenesis. Cell Death Differ. 2006;13: 1950–1959. doi:10.1038/sj.cdd.4401892
3. Pauli A, Althoff F, Oliveira RA, Heidmann S, Schuldiner O, Lehner CF, et al. Cell-Type-Specific TEV Protease Cleavage Reveals Cohesin Functions in Drosophila Neurons. Dev Cell. 2008;14: 239–251. doi:10.1016/j.devcel.2007.12.009
4. Weaver LN, Drummond-Barbosa D. The nuclear receptor seven up functions in adipocytes and oenocytes to control distinct steps of Drosophila oogenesis. Dev Biol. 2019;456: 179–189. doi:10.1016/j.ydbio.2019.08.015
5. Holtzman S, Miller D, Eisman R, Kuwayama H, Niimi T, Kaufman T. Transgenic tools for members of the genus drosophila with sequenced genomes. Fly (Austin). 2010;4: 349–362. doi:10.4161/fly.4.4.13304
6. Goda T, Doi M, Umezaki Y, Murai I, Shimatani H, Chu ML, et al. Calcitonin receptors are ancient modulators for rhythms of preferential temperature in insects and body temperature in mammals. Genes Dev. 2018;32: 140–155. doi:10.1101/gad.307884.117
7. Thibault ST, Singer MA, Miyazaki WY, Milash B, Dompe NA, Singh CM, et al. A complementary transposon tool kit for Drosophila melanogaster using P and piggyBac. Nat Genet. 2004;36: 283–287. doi:10.1038/ng1314
8. Parks AL, Cook KR, Belvin M, Dompe NA, Fawcett R, Huppert K, et al. Systematic generation of high-resolution deletion coverage of the Drosophila melanogaster genome. Nat Genet. 2004;36: 288–292. doi:10.1038/ng1312
9. Lajeunesse DR, Johnson B, Presnell JS, Catignas KK, Zapotoczny G. Peristalsis in the junction region of the Drosophila larval midgut is modulated by DH31 expressing enteroendocrine cells. BMC Physiol. 2010;10: 14. doi:10.1186/1472-6793-10-14
10. Bellen HJ, Levis RW, Liao G, He Y, Carlson JW, Tsang G, et al. The BDGP gene disruption project: Single transposon insertions associated with 40% of Drosophila genes. Genetics. 2004;167: 761–781. doi:10.1534/genetics.104.026427

11. Head LM, Tang X, Hayley SE, Goda T, Umezaki Y, Chang EC, et al. The influence of light on temperature preference in Drosophila. Curr Biol. 2015;25: 1063–1068. doi:10.1016/j.cub.2015.02.038

12. Ryder E, Ashburner M, Bautista-Llacer R, Drummond J, Webster J, Johnson G, et al. The DrosDel deletion collection: A Drosophila genomewide chromosomal deficiency resource. Genetics. 2007;177: 615–629. doi:10.1534/genetics.107.076216

13. Renn SCP, Park JH, Rosbash M, Hall JC, Taghert PH. A pdf neuropeptide gene mutation and ablation of PDF neurons each cause severe abnormalities of behavioral circadian rhythms in Drosophila. Cell. 1999;99: 791–802. doi:10.1016/S0092-8674(00)81676-1

14. Perkins LA, Holderbaum L, Tao R, Hu Y, Sopko R, McCall K, et al. The transgenic RNAi project at Harvard medical school: Resources and validation. Genetics. 2015;201: 843–852. doi:10.1534/genetics.115.180208

15. Pastor-Pareja JC, Xu T. Shaping Cells and Organs in Drosophila by Opposing Roles of Fat Body-Secreted Collagen IV and Perlecan. Dev Cell. 2011;21: 245–256. doi:10.1016/j.devcel.2011.06.026

16. Dietzl G, Chen D, Schnorrer F, Su KC, Barinova Y, Fellner M, et al. A genome-wide transgenic RNAi library for conditional gene inactivation in Drosophila. Nature. 2007;448: 151–156. doi:10.1038/nature05954
