# Supplementary material for: RNAi-based screens uncover a potential new role for the orphan neuropeptide receptor Moody in Drosophila female germline stem cell maintenance
Source: PLoS One. 2020 Dec 11;15(12):e0243756. doi: 10.1371/journal.pone.0243756 (PMC7732368; doi:10.1371/journal.pone.0243756)
Supplement: S2 Table — (PDF) [file pone.0243756.s010.pdf]

S2 Table. Sequences of primers used in this study.

Gene	Forward	Reverse
Dh31 **(Fig. 3)**	5'-ATGCCCAGGTACCAATCCAA-3'	5'-CAAAGCGAGTCATCAGTTCC-3'
Dh31 **(Fig. S3)**	5'-TCCTGTGAACGTTGAGGACG-3'	5'-TTGATCGTGTCCGACTCACG-3'
Dh31-R **(Fig. 3)**	5'-TTACGGCCTCATTTCAAGG-3'	5'-CCTGAGTGGCTGTATAGGA-3'
Dh31-R **(Fig. S3)**	5'-CGTTCGCTGCCAATAACTCG-3'	5'-ACGAGACCTGAGTGGCTGTA-3'
moody	5'- CAAGACGGTGGTTTTCTGTC-3'	5'-CTCAACCCGGTATCTTTCCA-3'
Rp49	5'-CAGTCGGATCGATATGCTAAGC-3'	5'-AATCTCCTTGCGCTTCTTGG-3'
