# Supplementary material for: RNAi-based screens uncover a potential new role for the orphan neuropeptide receptor Moody in Drosophila female germline stem cell maintenance
Source: PLoS One. 2020 Dec 11;15(12):e0243756. doi: 10.1371/journal.pone.0243756 (PMC7732368; doi:10.1371/journal.pone.0243756)
Supplement: Attachment — Submitted filename: PONE_D_20_32229_ResponseToReviewers.pdf [file pone.0243756.s012.pdf]

PONE-D-20-32229 - RESPONSE TO REVIEWERS

REVIEWER 1

Reviewer 1 acknowledged: ***"The study was performed with great care to detail and a lot of data were generated. Several RNAi lines were tested, as well as mutants, and for many of them RNA levels were determined. The conclusions are generally well supported by the results. (…) It is valuable to show the pitfalls of this approach in studying oogenesis, especially since the studies are done very carefully. Potential reasons and conclusions are well described."***

He/She also said that a few points should be clarified, as detailed below.

Point 1: ***"Fig. 2I: A description of what ApopTag labels would be useful in the figure legend. An arrow pointing out differences would be useful. I fail to see a difference between the control and the mutant. A better example for the observed difference is needed."***

We added a sentence in the figure legend explaining that dying cysts are labeled by ApopTag and found in both control and *Dh31-R* knockdown germaria, and these dying cysts look similar based on ApopTag staining (lines 178-179 of "clean" manuscript or lines 195-196 of manuscript with track changes).

Point 2: **"*Please describe what the "maternal triple drive MTD is.*"**

We have included information about the three Gal4 drivers that make up *MTD* (lines 109-110 or "clean" manuscript or lines 120-121 of manuscript with track changes).

Point 3: **"*For Dh3151 /Pdf01 double mutants the authors say that the reduced number of eggs laid might be due to egg retention. As evidence, they show the pictures of dissected ovaries and say that egg appendices can be seen in the mutants. This is not visible in the picture and a magnification of these ovaries should be shown, with arrows pointing to these structures."***

We have added more arrows to S5C Fig and made sure they point directly at the dorsal appendages of multiple examples of accumulated mature oocytes to help readers visualize these structures.

Point 4: **"*For moody, the authors say that in a separate screen they found reduced numbers of GSCs. This result is listed in the included table, but no data are shown. Furthermore, the authors say that in a later experiment with larger sample size, this could not be repeated. Since data are not shown, and could not be repeated, these findings should not be discussed as supporting evidence, but as a caveat for the RNAi findings."***

We initially identified *moody* in a separate screen, where knockdown of *moody* appeared to both reduce the number of GSCs and increase the percentage of EdU-positive GSCs. Later experimentation with larger sample sizes showed that while the reduction in GSC number was reproducible (Fig 3D-F), we no longer saw any statistically significant increase in the percentage of EdU-positive GSCs (S7 Fig). Results from the screen are described in lines 251-253 (of manuscript with track changes), and follow-up

experiments for EdU incorporation are described in lines 253-255 (of manuscript with track changes). We have added "EdU incorporation" in line 254 (of manuscript with track changes) to clarify the nature of these experiments, and implications of these findings (the requirement of larger sample sizes to accurately capture changes in GSC proliferation) are discussed in lines 397-404 (of manuscript with track changes).

<u>Point 5</u>: "***The authors mention that it will be interesting to study the downstream pathway of moody signaling. In development, there is evidence that this involves cAMP and PKA signaling. Is it known whether these pathways are needed for oogenesis, and in which cells?***"

PKA and cAMP signaling has been shown to be required in oocytes for microtubule cytoskeleton to reorganize properly during mid-oogenesis (Steinhauer and Kalderon, Development, 2005). However, there is no evidence in the literature that PKA signaling regulates GSC number.

<u>REVIEWER 2</u>

Reviewer 2 stated: ***"In this study, Ma and Drummond-Barbosa use RNAi-based approaches to screen for novel neural regulators of reproductive output (egg production). Unfortunately, their screens only identified a few potential regulators. However, the data collection is solid, the writing is top-notch, and publication of the data may form the foundation for other, more targeted studies in the future. Importantly, Ma and Drummond-Barbosa show that the neuropeptide receptor Moody is necessary in the soma for germline stem cell number, although the mechanisms by which this occurs remain to be elucidated. Given that this is the report of a genetic screen, the descriptive nature of the study is appropriate. The experiments are technically rigorous and the results described sufficiently. Overall, the text is well-written, and the conclusions are supported by the data presented."***

He/She had minor comments for revision, addressed below.

<u>Point 1</u>: ***"Results, lines 196-199. It is worth mentioning in the results/discussion of Dh31 and Pdf single and double mutants that the defects in egg laying or egg retention may suggest roles in ovulation, vs roles in egg production per se."***

We have added a sentence in lines 225 (of manuscript with track changes) potentially suggesting a role for *Dh31* and *Pdf* in ovulation.

<u>Point 2</u>: ***"Results, lines 282-285. The authors bring up insulin signaling to cap cells as a possible connection for why Moody mutants have fewer GSCs. This seems like a stretch to me. It is perhaps more reasonable to think that Moody, as a GPCR, is expressed in ovarian somatic cells (cap cells or otherwise) and thus regulates GSC number…maybe not affecting cap cell number, but rather cap cell interconnectivity or interaction with GSCs. It looks like a UAS-moody-RFP and a moody-Gal4 have been published elsewhere (Schwabe et al., Cell, 2005, 123(1):133-44). If possible, the authors should test whether Moody is expressed in ovarian somatic cells."***

Insulin signaling, which has a well-known role in regulating GSC number, is dependent on signals such as Unpaired 2 (Upd2) traveling past the blood-brain barrier to insulin-producing cells (IPCs) in the brain. Since Moody plays an essential role in maintaining the blood-brain barrier, we feel that it was reasonable to rule out if the GSC loss phenotype was due to global insulin signaling being disrupted due to *moody* knockdown. Regardless, our results indicated that GSC loss was not due to changes in insulin signaling, and we explored whether moody is required in somatic cells in the germarium using *hh-Gal4* (and found that it is not). (See lines 327-332 of manuscript with track changes.)

<u>Point 3</u>: "*Since most of the paper describes the RNAi screens, I am hesitant to suggest additional experiments. However, if the authors wanted to pursue Moody to more deeply understand why it is necessary in somatic cells to maintain GSC numbers, it would be interesting to use additional somatic drivers in the ovary and ovarian muscle sheath to test whether Moody impacts actin-dependent septate junction maintenance (either in cap cells or escort cells). This has been demonstrated for Moody in the maintenance of the blood brain barrier, and similar septate junction proteins (like Coracle, for example) are expressed in escort cells. A stronger somatic driver (like c587-Gal4) or one more specific to anterior escort cells (like Pdk1-Gal4) might also better recapitulate the tubulin-Gal4 finding than hh-Gal4, which is relatively weak.*"

We agree that it would be interesting in future studies to pursue *moody* using additional somatic drivers, both in the ovary and in other tissues to determine where *moody* is required for GSC maintenance and the mechanism through which *moody* regulates GSC numbers (whether through regulating septate junction proteins or other downstream pathways).

<u>Point 4</u>: *"Figure 3, panel I: there is a small asterisk at the 15d timepoint, but it is unclear to me whether this is indicating statistical significance? The timepoints seem to overlap?"*

There is an asterisk in Panel I for $moody^{KK100674}$, indicating statistical significance as determined by two-way ANOVA with replication. This method measures how two independent variables (in this case, genotype and time) interact to affect the dependent variable (the number of GSCs) and takes into account the slope of the curve. The statistical significance likely reflects the difference in slopes between $moody^{KK100674}$ and $Luc^{JF01355}$ from days 5 to 10 and from 10 to 15, though, as the reviewer points out, the overall slope of the two lines (from days 0 to 15) are very similar, which supports our conclusion that *moody* is likely not required in the niche for GSC maintenance.

<u>ACADEMIC EDITOR/JOURNAL REQUIREMENTS:</u>

"Please ensure that your manuscript meets PLOS ONE's style requirements, including those for file naming."

We have updated our manuscript formatting and file names to meet PLOS ONE's style requirements.

"*While revising your submission, please upload your figure files to the Preflight Analysis and Conversion Engine (PACE) digital diagnostic*

***tool, https://pacev2.apexcovantage.com/. PACE helps ensure that figures meet PLOS requirements.*"**

We have done so.
